# Notes and News

**Published:** 1903-09-05

**Authors:** 


### Notes and News.

Mr. R. Fox Symons, M.R.C.S., Acting Medical Officer of Health for the Transvaal, has been appointed Inspector-General of Health for the Transvaal.

Mr. Otto Hehner, a public analyst, draws the attention of the Royal Commission on Arsenical Poisoning to the use of oxide of iron as a colouring agent for foodstuffs. He states that he has detected arsenic in small quantities in most cases where the oxide has been used.

It is reported that the Holbeach Board of Guardians are about to purchase an old railway carriage to be prepared for use as an isolation hospital in case of need. This prompt and public-spirited action is said to have been suggested by the presence of small-pox at Cambridge.

The residue of the Manchester Queen Victoria Memorial Fund has been handed over to the Royal Infirmary, after the expenditure of £5,500 upon a statue of the Queen. The subscribers hope that the money, amounting to £13,000, will be utilised in some manner as to form a distinct memorial to Queen Victoria in the new buildings at Stanley Grove.

The subjects before the International Congress of Hygiene and Demography now being held in Brussels are of considerable interest. The subject of hygiene gains a greater importance every year, and consequently more leading scientists and larger audiences are being attracted to these congresses, from which it is always hoped that a fresh impetus may be given to important discoveries.

The repeated floods which have visited nearly all parts of the globe this year will with little doubt be responsible for a considerable amount of illness. The majority of drainage systems are not arranged to meet the tremendous tax placed upon them recently, and a large amount of unwholesome matter must also have been left on the surface in many places ready to breed disease when the process of evaporation commences. Already we learn that a serious outbreak of typhoid has occurred in Silesia, where the floods have been prevalent.

Last week there were no fresh cases of small-pox reported at Cambridge.

The epidemic of plague in Mauritius, though numerically small, is of a very fatal character.

An extraordinary prevalence of tuberculosis has come to light as existing at the Leavesden Asylum, Hertfordshire. In 1900 the deaths from this form of disease equalled 105, being one-third of the total number of deaths occurring at the institution. After this hygienic improvements were made, and the fatalities fell to 42 in 1902.

In view of the great loss which butchers incur by the purchase of animals affected with tuberculosis a conference of butchers has been held in Wales and a resolution put to the meeting which provides for a guarantee from vendors of animals, certifying them free from tuberculosis or other disease. The resolution has been put into the hands of a committee who will report upon its proposals later.

A visit to the Finsen Institute at Copenhagen made by a member of our staff revealed the fact that cases which had undergone the light cure in London without being cured have gone to Copenhagen and found the treatment efficacious. An impression prevails that the treatment is not at present properly understood in London, and that it would be an advantage if more nurses in charge of Light Departments in London could be sent to the Finsen Institute in Copenhagen and thoroughly trained for the work. The Danish doctors appear to pay very close individual attention to each case.

A protest from a medical man, which seems to be quite reasonable, has arisen out of the provisions of the new Midwives Act. He points out that when a midwife calls in a medical man in cases where complications arise, that some special fee should be forthcoming. Seeing that the midwife is likely to monopolise all simple cases, it seems hard that in cases of difficulty and greater responsibility the doctor should supplement the services of the midwife and in all probability be expected to do so for a small fee or none. Many practitioners would feel inclined to forego their fee from poor patients when they feel that the midwife's fee has also to be met.

Through a correspondence with the Duke of Northumberland, as representing the Imperial Vaccination League, the National Anti-vaccination League has drawn public attention to the important fact that the late small-pox epidemic in London cost £500,000 for accommodation alone. This fact should not be forgotten by the more intelligent members of the community when an opportunity occurs to urge the advantages of vaccination as a preventive measure, if only on the score of economy. In this connection the report of the Metropolitan Asylums Board for 1902 is very interesting. Commenting upon the small-pox epidemic, it is pointed out that the percentage of deaths amongst children under 10 years of age in the case of vaccinated children was 1·4 per cent., and amongst the unvaccinated 34·7. Amongst the whole number of patients the percentage of mortality was 7·3 per cent. amongst the vaccinated and 33 1 per cent. amongst the unvaccinated.

